# Effect of physical activity on body condition in Swedish companion dogs

**DOI:** 10.1186/1751-0147-57-S1-P10

**Published:** 2015-09-25

**Authors:** Sara Ringmark, Emma Björk, Anna Jansson

**Affiliations:** 1 Department of Animal Nutrition and Management, Swedish University of Agricultural Science, Uppsala, Sweden

## Introduction

Obesity is believed to be an increasing health and welfare problem among companion dogs. In general, obesity is the result of imbalanced energy intake and utilisation, i.e. too much feed in relation to level of physical activity.

## Objectives

The aim of this study was to assess body condition among Swedish companion dogs and to study if it could be associated with type and amount of exercise performed.

## Material and methods

Body condition score (BCS, scale 1-9) was assessed in 102 companion dogs (inclusion criteria 1-10 years, max 120 min/week of activities in addition to walks) and owners were interviewed about daily time of walks, activity level of the dog during walks (Low: mostly walk, Medium: trot and some running, High: running) and of additional activities (yes/no). Statistical analysis were performed using a mixed model including age (≤5 years/>5 years) activity level and additional activity as fixed effects and minutes of walk/day as a continuous variable. Effects were considered significant at p<0.05 and values are presented as lsmeans ± SE.

## Results

Thirty dogs (29%) had a BCS ≥6 and accordingly described as fat or obese. The BCS decreased as the level of activity during daily walks increased (Low 5.7 ± 0.3, Medium 4.7 ± 0.2, High 3.3 ± 0.3, p<0.0001) and BCS was also lower in dogs performing additional activities (3.9 ± 0.3 vs. 5.2 ± 0.2). Minutes of walks/day did not affect BCS.

## Conclusion

The study indicates that activity level during walks and additional activities may have a greater impact on BCS than duration of walks.

